# Loss of *dihydrolipoyl succinyltransferase* (DLST) leads to reduced resting heart rate in the zebrafish

**DOI:** 10.1007/s00395-015-0468-7

**Published:** 2015-02-20

**Authors:** Mirjam Keßler, Ina M. Berger, Steffen Just, Wolfgang Rottbauer

**Affiliations:** Department of Medicine II, Cardiology, University of Ulm Medical Center, Albert-Einstein-Allee 23, 89081 Ulm, Germany

**Keywords:** Bradycardia, Pacemaker cell function, Mitochondria, *Dihydrolipoyl succinyltransferase*, ATP

## Abstract

The genetic underpinnings of heart rate regulation are only poorly understood. In search for genetic regulators of cardiac pacemaker activity, we isolated in a large-scale mutagenesis screen the embryonic lethal, recessive zebrafish mutant *schneckentempo* (*ste*). Homozygous *ste* mutants exhibit a severely reduced resting heart rate with normal atrio-ventricular conduction and contractile function. External electrical pacing reveals that defective excitation generation in cardiac pacemaker cells underlies bradycardia in *ste* ^−/−^ mutants. By positional cloning and gene knock-down analysis we find that loss of *dihydrolipoyl succinyltransferase* (DLST) function causes the *ste* phenotype. The mitochondrial enzyme DLST is an essential player in the citric acid cycle that warrants proper adenosine-tri-phosphate (ATP) production. Accordingly, ATP levels are significantly diminished in *ste* ^−/−^ mutant embryos, suggesting that limited energy supply accounts for reduced cardiac pacemaker activity in *ste* ^−/−^ mutants. We demonstrate here for the first time that the mitochondrial enzyme DLST plays an essential role in the modulation of the vertebrate heart rate by controlling ATP production in the heart.

**Electronic supplementary material:**

The online version of this article (doi:10.1007/s00395-015-0468-7) contains supplementary material, which is available to authorized users.

## Introduction

Diseases of cardiac pacemaker cells such as the sick sinus syndrome are the most common indications for cardiac pacemaker implantations [12, 27]. However, little is known about the genetic underpinnings of cardiac pacemaker function in vertebrates. Genetic studies in humans have demonstrated a significant contribution of genetic factors in heart rate regulation [11, 12, 38]. For instance, it was found that autosomal recessive mutations of the sodium-channel SCN5A lead to familial sick sinus syndrome [4]. Furthermore, a combined genome-wide association study that included data from 181,171 individuals recently led to the identification of 14 distinct genomic regions that significantly impact on heart rate regulation [12]. Interestingly, in these regions both, genes already known to be involved in heart rate regulation such as the hyperpolarization-activated cyclic nucleotide-gated cation channel 4 (*HCN4*), but also several novel modifier genes are located [12].

In recent years, large-scale forward genetic screens in zebrafish helped to dissect novel genetic underpinnings of cardiac rhythm control [10, 20, 31, 34, 42]. For instance, characterization of the zebrafish mutants *island beat* and *tremblor* revealed an essential role of the L-type calcium channel alpha 1 subunit and the sodium–calcium exchanger NCX1h in the pathogenesis of atrial and ventricular fibrillation, respectively [15, 24, 37]. Furthermore, characterization of a bradycardic zebrafish mutant and gene knock-down studies recently revealed an essential role of Shox2-Islet1 signaling in the control of cardiac pacemaker activity [21].

In search for novel regulators of basal heart rate we dissected here the genetic cause of the embryonic-lethal recessive ethylnitrosourea (ENU)-induced bradycardic zebrafish mutant *schneckentempo* (*ste*), and find that DLST, a mitochondrial enzyme that acts within the citric acid cycle to warrant ATP production, is essential for cardiac pacemaker cells.

## Materials and methods

Zebrafish (*Danio rerio*) were bred and maintained at 28.5 °C as described by Westerfield [47]. Pictures and movies were recorded at 48 h post-fertilization (hpf) and 72 hpf. To inhibit pigmentation 0.003 % 1-phenyl-2-thiourea was added to the regular embryo medium E3 (5 mM NaCl, 0.17 mM KCl, 0.33 mM CaCl_2_, 0.33 mM MgSO_4_ dissolved in water). Heart rate was counted at 48, 72, 96 and 120 hpf at room temperature.

We measured fractional shortening by videomicroscopy at room temperature and compared the diameters of the ventricle at the end of contraction (systole) and relaxation (diastole) as described before [31].

DNA from 48 *ste* ^−/−^ mutant and 48 wild-type embryos was pooled and bulked segregation analysis was performed as described before [33]. The critical genomic interval for *ste* was defined by genotyping 613 mutant embryos for polymorphic markers in the area. RNA from *ste* ^−/−^ mutant and wild-type embryos was isolated using TRIZOL Reagent (Life Technologies) and reverse transcribed. cDNA of a total of 30 mutant and wild-type embryos was sequenced. Genomic DNA from ten *ste* ^−/−^ mutant and ten wild-type embryos was sequenced around the point mutation.

Quantitative real-time PCR was carried out according to standard protocols with the SYBR-Green method (Bio-Rad) and an Eppendorf Realplex-2 cycler. cDNA was generated from RNA of 72 hpf homozygous *ste* mutant and sibling embryos using oligo(dT) primer and SuperScript III reverse transcriptase (Invitrogen).

For all Morpholino-modified antisense oligonucleotide injection procedures, the TE wild-type strain was used. Morpholino-modified antisense oligonucleotides (MO; Gene Tools, LLC) were directed against the translational start site (5′-TCAGACAGCGGGAATGACACAACAT-3′) of zebrafish DLST (MO-DLST-start), the splice donor site of exon 5/intron 5 (5′-CATGTTGCCTTCTTACCTTTCTCCC-3′) of zebrafish DLST on chromosome 17 (MO-DLST-splice), the splice donor site exon 3/intron 3 (5′-ACTGGATGTCAGATCGGTACCTTAA-3′) of DLDH on chromosome 25 (MO-DLDH-splice), the splice donor site exon 5/intron 5 of zebrafish OGDH a (5′-AGACCTTCAAATCTTCTACCTGTGC-3′) on chromosome 8 (MO-OGDHa-splice) and the splice donor site exon 7/intron 7 of zebrafish OGDH b (5′-TTCTTGTTGTCCTGACTTACCTCTA-3′) on chromosome 10 (MO-OGDHb-splice). DLST, OGDH and DLDH Morpholino-modified antisense oligonucleotides or a standard control Morpholino (MO-control) were microinjected into zebrafish embryos up to the 4-cell stage.

For histology, embryos were fixed with 4 % paraformaldehyde and embedded in JB-4 (Polysciences, Inc). Then, 5-μm sections were cut, dried, and samples were stained with hematoxylin and eosin. Transmission electron micrographs (TEM) were obtained essentially as described previously [37]. Whole-mount antisense RNA in situ hybridization was carried out as described previously [22, 23] using a digoxigenin-labeled antisense probe for zebrafish DLST.

Calcium imaging was performed as described before [28]. Wild-type and *ste* ^−/−^ mutant embryos were injected with 1 nl of a 250 μM stock solution of calcium green-1 dextran (Molecular Probes) at the one-cell stage. At 72 hpf videos were recorded with a Proxitronic (Proxivision) camera at 29.97 frames per second. Relative fluorescence of the atrium and ventricle were analyzed with custom-made software [31].

Electrical stimuli were applied by a pacemaker (PACE100H; Osypka Medical GmbH). Patch clamp pipettes filled with 3 M potassium chloride with a resistance of 2–10 MΩ were used as cathode. As described before [31, 37], this electrode was approached to either the atrium or ventricle with a micromanipulator under microscopic control. The heart chambers were stimulated at different frequencies (80–150/min), applying 1–12 V.

Sodiumfluoroacetate (SFA) (FCH_2_CO_2_Na) was added at a concentration of 0.067 or 0.2 mg/ml to the medium of dechorionated wild-type zebrafish at 24 hpf and exchanged every 24 h. SFA blocks aconitate hydratase thereby inhibiting the citric acid cycle [36].

2,4-Dinitrophenol (DNP) (Sigma Aldrich) was added at a concentration of 10 µM to the medium of dechorionated wild-type zebrafish embryos at 48 hpf. 2,4-Dinitrophenol uncouples the respiratory chain reaction from ATP production [8, 39, 46]. At high concentrations it is known to decrease ATP production [9, 46], whereas at lower concentrations predominantly reactive oxygen species (ROS) production is influenced [8, 39].

Idebenone (IDB, Sigma Aldrich) proved to act antioxidative against ROS and to promote mitochondrial function and ATP production after acute exposure by increasing the effectiveness of the respiratory chain reaction [6, 19]. Zebrafish embryos were incubated at 48 hpf at a concentration of 0.3 µM dissolved in ethanol.

ATP content was measured with a luciferase-based assay (ATP Determination Kit A22066, Invitrogen, Life Technologies Corporation) with recombinant firefly luciferase and d-luciferin. Zebrafish embryos were mechanically lysed and luminescence was immediately quantified by Berthold Luminometer Lumat LB 9501. The ATP content was calculated by creating an ATP-luminescence standard curve in Microsoft Excel.

Data of protein expression was obtained from the protein expression database MOPED (http://www.proteinspire.org/MOPED/mopedviews/proteinExpressionDatabase.jsf) and PaxDb (http://www.pax-db.org).

All results are depicted as arithmetic mean ± standard error of mean from at least two independent experiments. Figures were created with Microsoft Excel. Area measurements in TEM were conducted with ArchiCAD (Graphisoft).

Statistical analysis was performed in Graph Pad Prism. Comparisons between experimental groups were performed using *t* test. Differences are considered significant at a *p* < 0.05 significance level and marked with “*”, very significant with *p* < 0.01 are labeled “**” and extremely significant with *p* < 0.001 are labeled “***”.

## Results

### Zebrafish mutant *schneckentempo* exhibits bradycardia

In search for genetic modulators of the vertebrate heart rate, we isolated in a large-scale ENU-mutagenesis screen (Tübingen 2000) the embryonic-lethal zebrafish mutant line *schneckentempo* (*ste*), which displays a severely reduced resting heart rate, so-called bradycardia. Besides their reduced heart rate, *ste* ^−/−^ mutant zebrafish embryos exhibit decreased motility both spontaneously and in response to touch.

**Fig. 1:**
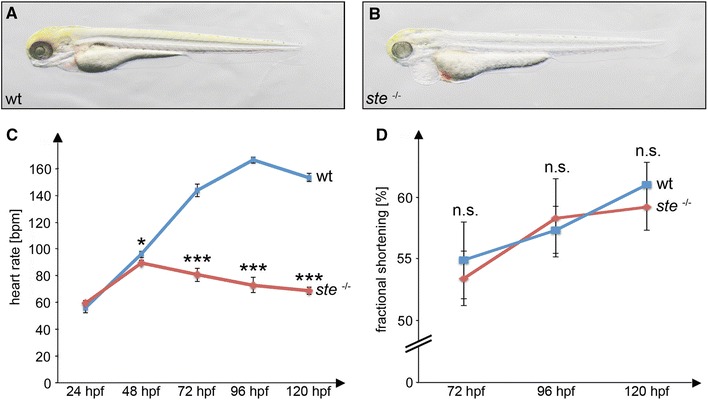
Effects of the *ste* mutation on cardiac function. **a, b** Lateral view of wild-type (wt) (**a**) and *ste* ^−/−^ mutant (**b**) embryos at 72 h post-fertilization (hpf). *ste* ^−/−^ mutants (**b**) develop blood congestion at the cardiac inflow tract and a pericardial edema. **c** Heart rate at different time points in *ste* ^−/−^ mutants (*red*) and wild-type littermates (*blue*). Bradycardia in *ste* ^−/−^ mutants starts at 48 hpf and becomes more severe during further embryonic development. **d** Ventricular fractional shortening is unaffected in *ste* ^−/−^ mutants compared to wild-type littermates at 72 hpf (53 ± 2.2 vs. 55 ± 3.1 %, *n* = 5, *p* = 0.7096), 96 hpf (58 ± 3.2 vs. 57 ± 1.9 %, *n* = 5, *p* = 0.7956) and 120 hpf (59 ± 1.9 vs. 61 ± 1.8 %, *n* = 5, *p* = 0.5586)

Up to 72 h post-fertilization (hpf), *ste* ^−/−^ mutant embryos are indistinguishable from their wild-type littermates in regard to cardiac structure and function. However, at 72 hpf *ste* ^−/−^ mutant embryos display severe bradycardia with a heart rate of 81 ± 4.7 beats per minute (bpm) compared to 143 ± 4.7 bpm in wild-type siblings (*n* = 16, *p* < 0.0001) measured at room temperature. During further development the heart rate of *ste* ^−/−^ mutant embryos even further decreases at 96 hpf to 73 ± 2.3 bpm compared to 167 ± 5.7 bpm (*n* = 16, *p* < 0.0001) and to 68 ± 6.5 bpm compared to 163 ± 3.9 bpm in wild-type siblings at 120 hpf (*n* = 16, *p* < 0.0001) (Fig. 1c). Although the heart rate is significantly reduced in *ste* ^−/−^ mutants, similar to the situation in wild-types, *ste* ^−/−^ hearts contract regularly and sequentially, whereby each atrial contraction is followed by a ventricular contraction, indicating unaffected atrio-ventricular conduction in *ste* ^−/−^ hearts. Similarly, cardiac contractility is not reduced in homozygous *ste* mutants. As shown in Fig. 1d ventricular fractional shortening is 53 ± 2.2 % in *ste* ^−/−^ mutants and 55 ± 3.1 % in wild-type *ste* siblings at 72 hpf (*n* = 5, *p* = 0.7096), a time point where severe bradycardia is already manifest in *ste* ^−/−^ mutant embryos.

### The *ste* locus encodes for zebrafish *dihydrolipoyl succinyltransferase*

**Fig. 2:**
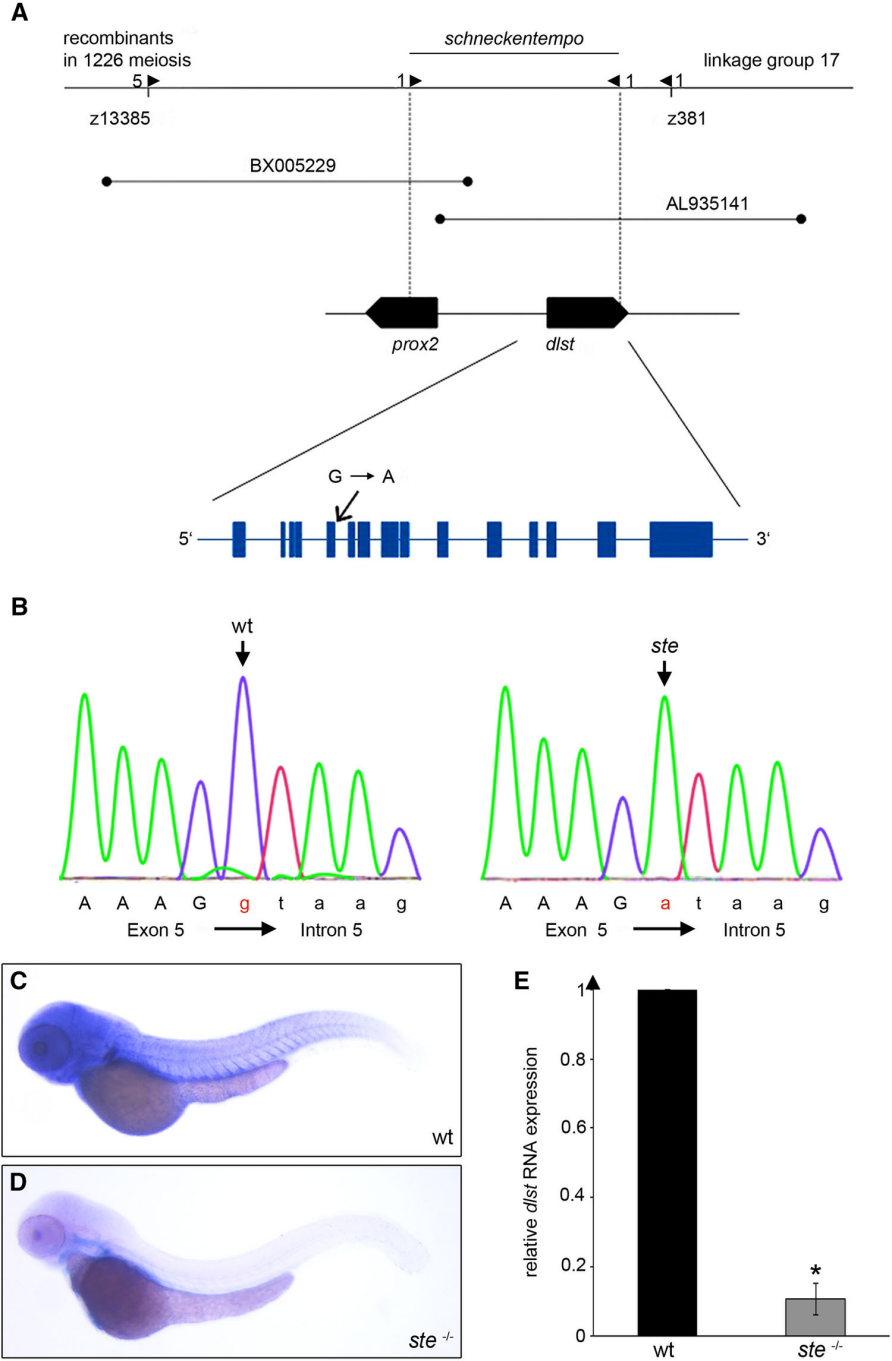
*ste* encodes for *dihydrolipoyl succinyltransferase* (*dlst*). **a** Integrated genetic and physical map of the *ste* locus. The *ste* mutation interval is flanked by the microsatellite markers z13385 and z381 and encodes two open reading frames, zebrafish *dlst* and *prox2*. **b** Within the coding sequence of *zdlst* a point mutation (G→A) was identified at the splice donor site of intron 5 leading to aberrant pre-mRNA splicing and the premature termination of DLST translation. An *arrow* marks the mutated base. **c**, **d** Spatial expression of *dlst by* whole-mount antisense RNA in situ hybridization of wt and *ste* ^−/−^ embryos at 72 hpf. **c** *dlst* is ubiquitously expressed with pronounced levels in the brain, skeletal muscle, fins and heart of wild-type embryos. **d** Strongly reduced *dlst* mRNA levels in *ste* ^−/−^ mutants compared to wild-type littermates. **e** Quantitative real-time PCR of *ste* ^−/−^ mutants and wild-type siblings. Nonsense mediated *dlst*-RNA decay in *ste* ^−/−^ mutants compared to wild-type siblings at 72 hpf

To identify the mutation causing the recessive *ste* ^−/−^ mutant phenotype, we performed a genome-wide study of microsatellite marker segregation and linked *ste* to zebrafish chromosome 17. Recombination analysis of 1,226 *ste* ^−/−^ mutant embryos restricted *ste* ^−/−^ to a genomic interval flanked by the microsatellite markers z13385 and z381. Genetic fine-mapping further narrowed down the *ste* locus to the two bacterial artificial chromosomes (BAC) BX005229 and AL935141, and finally to two open reading frames encoding proteins highly homologous to human *prospero*-*related homeobox 2* (*prox2*) and *dihydrolipoyl succinyltransferase* (*DLST*) (Fig. 2a). To identify the site of the ENU-induced mutation in *ste*, we sequenced the entire zebrafish coding sequence of the 2 genes z*prox2* and *zdlst* from wild-type and *ste* ^−/−^ mutant genomic DNA and identified the *ste* mutation to be a guanine to adenine nucleotide transition in the splice donor site of intron 5 of the z*dlst* gene (ENSDARG00000014230). *Zdlst* cDNA analysis of *ste* ^−/−^ mutants reveals that upstream of the mutated splice donor site an alternative splice site is used, resulting in partial exon skipping with deletion of 11 nucleotides (Fig. 2b). The consecutive reading frame shift generates an early stop codon and is predicted to cause premature termination of translation of *dlst* in *ste* ^−/−^ mutants (Supplementary Fig. 1). Next, to evaluate if the *ste* mutation impairs RNA stability, we quantified RNA levels by whole-mount RNA antisense in situ staining and qRT-PCR and find reduced levels of *dlst* RNA down to 10.7 ± 5.3 % in *ste* ^−/−^ mutants (*n* = 4, *p* = 0.0414) in comparison to wild-type *ste* siblings (Fig. 2e).

DLST is an essential component of the α-ketoglutarate dehydrogenase complex of the citric acid cycle and is expressed ubiquitously in mammals with pronounced levels in the liver, the kidneys, the central nervous system and the heart (databases for protein expression MOPED and PaxDb). As shown in Fig. 2c, we find *dlst* RNA to be ubiquitously distributed in wild-type zebrafish embryos, with enhanced levels in the heart, skeletal muscle and pectoral fins. Zebrafish DLST is highly homologous to human DLST with a 74 % overall amino acid identity (Supplementary Fig. 1). The *ste* mutation resides within the NH_2_-terminal lipoyl domain of DLST and hence the COOH-terminal catalytic domain of DLST is predicted not to be translated in *ste* ^−/−^ mutants leading to loss of DLST function.

**Fig. 3:**
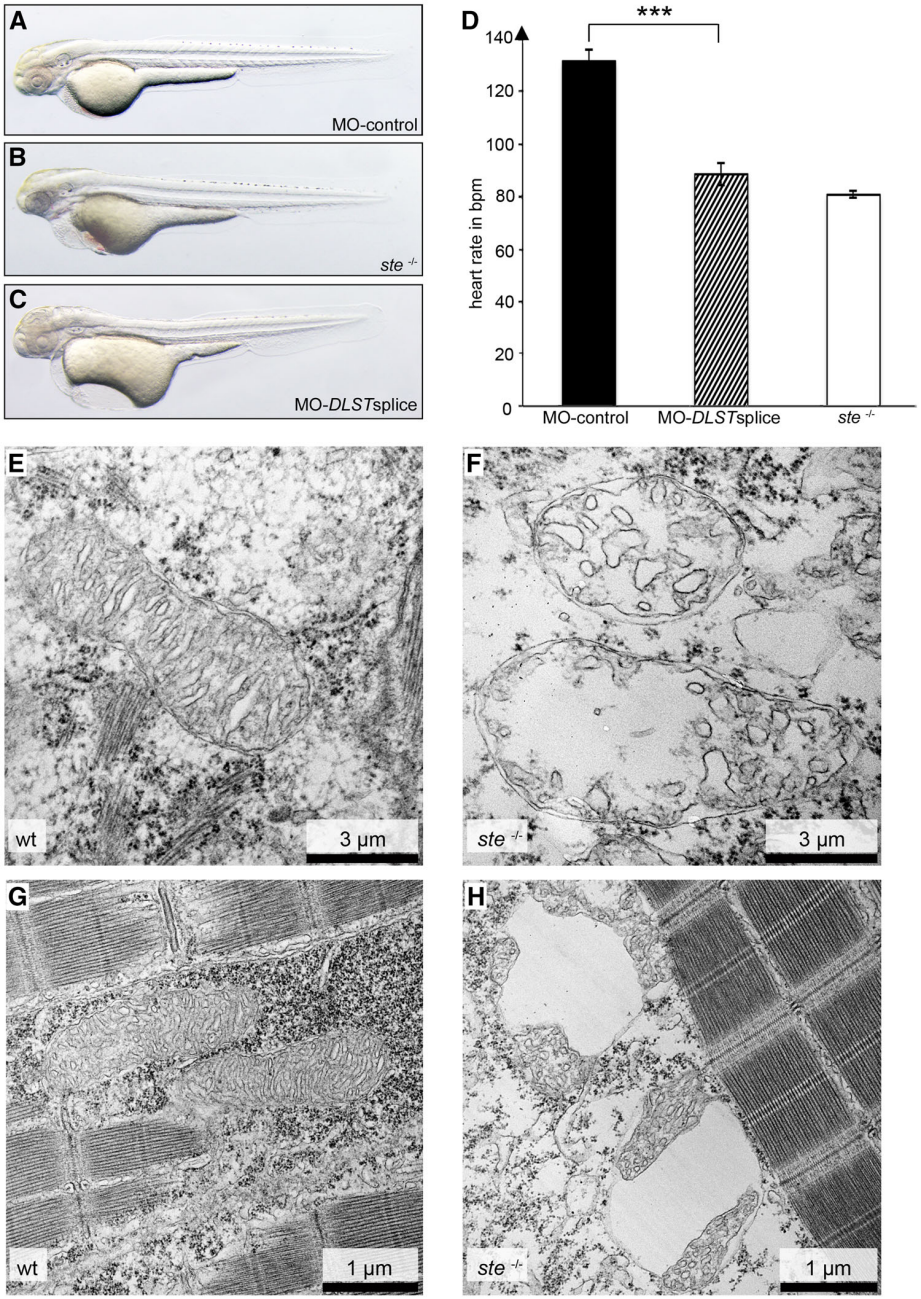
Targeted knockdown of z*dlst* phenocopies the *ste* ^−/−^ mutant phenotype. **a, b** Knockdown of *zdlst* by injection of Morpholino-modified antisense oligonucleotides (MO-*DLSTsplice*) (**a**) phenocopies the *ste* mutant phenotype (**b**), whereas injection of the same amount of standard control Morpholino (MO-control) (**c**) does not impact on heart rate. **d** Similar to *ste* ^−/−^ mutants, heart rate of *DLST*splice morphants is significantly reduced compared to MO-control-injected embryos at 72 hpf. **e**, **f** Transmission electron microscopy (TEM) of wild-type and *ste* ^−/−^ ventricular myocardium at 72 hpf. *Scale bar* represents 3 µM. **e** In wild-type myocardium, mitochondria display regular structure of inner and outer membrane and cristae. **f** In *ste* ^−/−^ myocardium, mitochondria display derangement of structure with severely reduced numbers of cristae. **g**, **h** TEM of wild-type and *ste* ^−/−^ skeletal muscle at 72 hpf. *Scale bar* represents 1 µM. **g** In wild-type skeletal muscle mitochondria exhibit regular morphology. **h** In *ste* ^−/−^ skeletal muscle mitochondria are characterized by large vacuoles and reduced numbers of cristae

To validate that loss of DLST function indeed is responsible for the *ste* phenotype, we inactivated *zdlst* by Morpholino-modified antisense oligonucleotide mediated gene knock-down, targeting either the translation initiation site (MO-*DLST*start) or the splice donor site of the exon 5/intron 5 boundary (MO-*DLST*splice) of z*dlst*, where the *ste* mutation resides. Injection of 4.2 ng MO-*DLST*splice into wild-type zebrafish embryos leads to bradycardia (89 ± 4 bpm) in 72 hpf embryos, whereas MO-control injected embryos exhibit normal heart rates of 132 ± 1 bpm (*n* = 14, *p* < 0.0001, Fig. 3d). Similar results were obtained by injection of 21.2 ng of MO-*DLST*start (heart rate at 72 hpf in MO-*DLST*start morphants 92 ± 6 bpm; in controls 139 ± 2 bpm; *n* = 10, *p* < 0.0001), indicating that the *ste* mutation leads to complete loss of zDLST function.

### Loss of DLST function induces mitochondrial degeneration in *schneckentempo* mutants

*Dihydrolipoyl succinyltransferase* is known to localize to the mitochondrial matrix where it catalyzes within the α-ketoglutarate dehydrogenase complex the citric acid cycle. Loss of mitochondrial enzyme function is often accompanied by morphological alterations of mitochondria [1, 7, 25, 45]. To determine if this is also the case in DLST-deficient *ste* ^−/−^ mutant zebrafish, we analyzed the mitochondrial ultrastructure in ventricular and atrial cardiomyocytes of *ste* ^−/−^ mutants by transmission electron microscopy (TEM). We find in *ste* ^−/−^ hearts large vacuoles either in the vicinity of mitochondria or within mitochondria. Their position is shifted from the interfibrillar to the sub-sarcolemmal and perinuclear regions as seen in other mitochondriopathies involving the heart [25]. Furthermore, similar to other mitochondriopathies [25] mitochondrial density is altered in *ste* ^−/−^ myocardium compared to wild-type embryos (2.20 ± 0.29 % in *ste* ^−/−^ versus 1.01 ± 0.20 % in wild-type, *n* = 100, *p* = 0.001) and the average size of mitochondria is significantly enlarged (0.67 ± 0.072 µm^2^ in *ste* ^−/−^ versus 0.35 ± 0.027 µm^2^ in wild types, *n* = 100, *p* < 0.0001). In addition, as shown in Fig. 3 mitochondria in *ste* ^*−*/−^ mutant hearts are pleomorphic, irregularly shaped and contain reduced numbers of cristae (4.8 ± 0.76 in *ste* ^−/−^ and 15.2 ± 1.7 in wild-type, *n* = 15, *p* = 0.0001). Similar changes on mitochondrial morphology could be observed in other *ste* ^−/−^ mutant tissues such as the skeletal muscle (Fig. 3g, h).

### Resting heart rate depends on the citric acid cycle

**Fig. 4:**
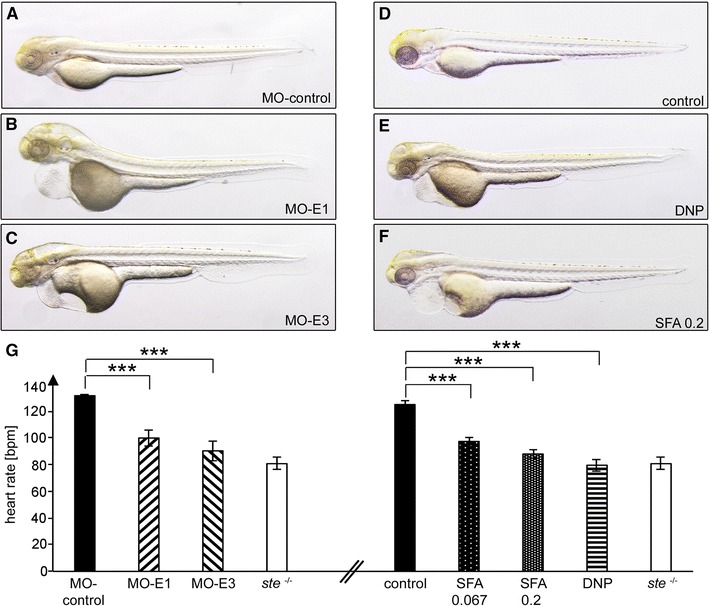
Blockade of the citric acid cycle phenocopies the *ste* ^−/−^ mutant phenotype. **a**–**c**, **g** Morpholino-modified antisense oligonucleotide mediated knockdown of *OGDH* subunit E1 (MO-E1) (**a**) or *DLDH* subunit E3 (MO-E3) (**b**) of the citric acid cycle phenocopies the *ste* ^−/−^ bradycardia (**g**), whereas injection of a control Morpholino has no effect (**c**, **g**). **d**–**g** Incubation of wild-type embryos with the citric acid cycle blocker sodiumfluoroacetate (SFA) and the respiratory chain uncoupler 2,4-dinitrophenol (DNP) phenocopies the heart rate defect of *ste* ^−/−^ mutant embryos in a dose-dependent manner

The citric acid cycle guarantees energy production by metabolizing amino acids, fatty acids and carbon hydrates [13]. Within the citric acid cycle, the α-ketoglutarate dehydrogenase complex is the rate-limiting component [30] and consists of three subunits: E1—oxoglutarate dehydrogenase (OGDH), E2—*dihydrolipoyl succinyltransferase* (DLST) and E3—dihydrolipoyl dehydrogenase (DLDH) [5, 18, 26]. To evaluate if the effect of DLST on zebrafish heart rate is mainly mediated via the α-ketoglutarate dehydrogenase complex, we inactivated the other subunits of this complex by Morpholino-modified antisense oligonucleotide mediated knock-down in zebrafish. To do so, we first identified the zebrafish subunit E1 and subunit E3 orthologous sequences and designed corresponding Morpholino-modified antisense oligonucleotides to inactivate their function. When co-injecting 10.4 ng Morpholino-modified antisense oligonucleotides targeting the splice site of exon 5 of subunit E1*a* (MO-E1*a*) and 5.4 ng of MO-E1*b* targeting the splice site of exon 7 of subunit E1*b* into zebrafish wild-type embryos, 93 % (*n* = 40) of the injected embryos displayed significantly reduced heart rates at 72 hpf (100 ± 6 bpm, in controls 133 ± 2 bpm, *n* = 10, *p* < 0.0001) similar to homozygous *ste* ^−/−^ mutant embryos (Fig. 4e). Similarly, after injection of 21.2 ng of Morpholino-modified antisense oligonucleotide directed to the splice site of exon 3 of subunit E3 (MO-E3), severe bradycardia with 90 ± 7 bpm could be observed in 89 % (*n* = 18) of the injected embryos at 72 hpf, indicating that indeed loss of the α-ketoglutarate dehydrogenase complex activity of the citric acid cycle accounts for bradycardia in *ste* ^−/−^ mutants (Fig. 4e).

Finally, to evaluate whether basal heart rate is regulated via the citric acid cycle or merely the α-ketoglutarate dehydrogenase complex, we blocked the citric acid cycle by a pharmacological approach independent of the α-ketoglutarate dehydrogenase complex. To do so, we inhibited aconitate hydratase and thereby efficiently citric acid cycle function by sodiumfluoroacetate (SFA) [36]. After incubation of 24 hpf zebrafish wild-type embryos for 48 h in 0.2 mg/ml SFA 83 % (*n* = 30) of the wild-type embryos exhibit similar to *ste* ^−/−^ mutants bradycardia with an average heart rate of 78 ± 3 bpm compared to 125 ± 3 bpm in controls (*n* = 11, *p* < 0.0001). Remarkably, incubation of wild-type embryos in much lower doses of SFA (0.067 mg/ml) also impacts on heart rate, but with a less pronounced effect (heart rate 97 ± 3 bpm and 125 ± 3 bpm in controls; *n* = 11, *p* < 0.0001, Fig. 4e), demonstrating a dose-dependent effect of citric acid cycle inhibition on zebrafish heart rate. In summary, these findings indicate an essential role of the citric acid cycle in zebrafish heart rate control.

### Reduced ATP levels lead to cardiac dysfunction in *ste*^−/−^ mutant zebrafish

**Fig. 5:**
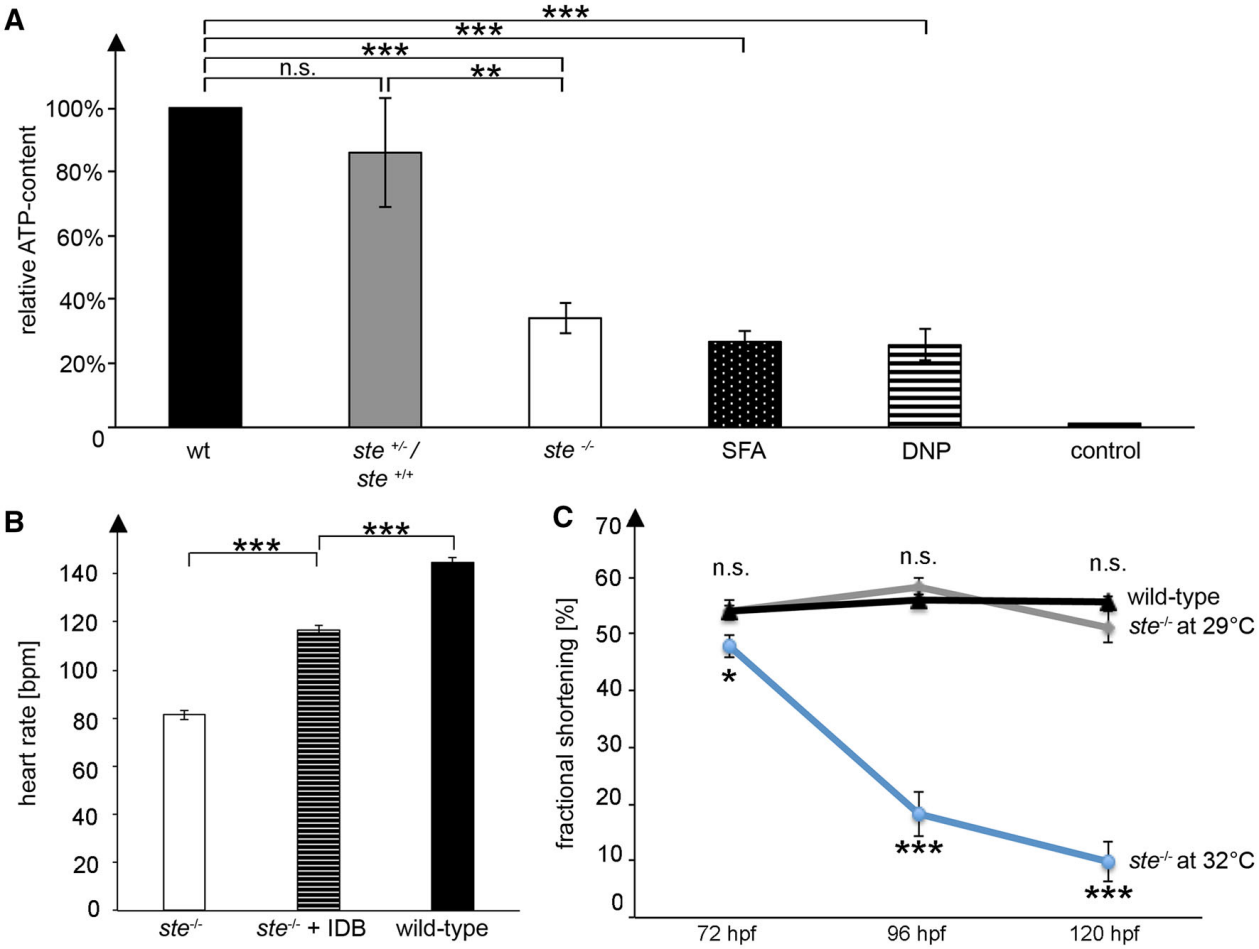
Reduced ATP levels in *ste* ^−/−^ mutants lead to sinus bradycardia. **a** ATP content of *ste* ^−/−^ mutant embryos is significantly reduced compared to wild-type embryos (TE strain) and wild-type *ste* siblings (*ste* ^+/−;+/+^ *)*, whereas ATP content between TE strain and wild-type *ste* siblings does not significantly differ. Dead zebrafish embryos serve as a control (72 hpf). The ATP content of embryos that were incubated with SFA and DNP is also significantly reduced and resembles the ATP content of *ste* ^−/−^ mutant embryos. **b** The coenzyme Q10 analogue Idebenone partially rescues heart rate in *ste* ^−/−^ mutant embryos that were incubated with this drug. **c** *ste* ^−/−^ mutants were incubated at increased temperature throughout embryonic development and ventricular fractional shortening was measured in wild-type and *ste* ^−/−^ mutant embryos at 72, 96 and 120 hpf. Fractional shortening of *ste* ^−/−^ mutant embryos under normal conditions (29 °C) does not significantly differ compared to wild-type embryos. Fractional shortening is reduced in *ste* ^−/−^ mutant embryos raised at 32 °C at (48 ± 2.1 vs. 54 ± 3.9 %, *n* = 10, *p* = 0.0358), 96 hpf (18 ± 4.0 vs. 56 ± 2.0 %, *n* = 10, *p* < 0.0001) and 120 hpf (10 ± 3.5 vs. 56 ± 2.1 %, *n* = 10, *p* < 0.0001)

The citric acid cycle is an essential metabolic process that ensures energy delivery under normoxic conditions. Within the citric acid cycle GTP (guanine-tri-phosphate), NADH (reduced nicotinamide adenine dinucleotide) and FADH_2_ (reduced flavin adenine dinucleotide) are produced and ultimately converted to ATP by the respiratory chain reaction [13]. Hence, to evaluate the impact of loss of DLST function on mitochondrial ATP production, we next measured ATP content in *ste* ^−/−^ mutants with a luciferase-based assay. As depicted in Fig. 5a, we find severely reduced levels of ATP in *ste* ^−/−^ mutants (34 ± 0.5 %, 75 ± 12 pmol) in comparison to wild-type zebrafish embryos (218 ± 32 pmol, *n* = 10, *p* = 0.0006) or wild-type *ste* siblings (86 ± 17 %, 188 ± 37 pmol, *n* = 10, *p* = 0.0096).

To further evaluate if indeed reduction of ATP directly accounts for bradycardia in *ste* ^−/−^ mutants, we next pharmacologically inhibited ATP production by incubation with 2,4-dinitrophenol (DNP) [8, 9, 46]. As shown in Fig. 5a, we find an average reduction of ATP levels in 72 hpf wild-type zebrafish embryos incubated for 24 h in 10 µM DNP down to 26 % of the ATP content of wild-type embryos (*n* = 10, *p* < 0.0001) accompanied by severe reduction of resting heart rate of DNP incubated embryos down to 80 ± 4 bpm compared to 125 ± 3 bpm in controls (*n* = 10, *p* < 0.0001). To further prove that the effect of loss of DLST on resting heart rate is mainly mediated via ATP, we incubated *ste* ^−/−^ mutant zebrafish embryos with the coenzyme Q10 analogue Idebenone (IDB), a known enhancer of global mitochondrial function and ATP production [6, 19]. As shown in Fig. 5b, incubation with 0.3 µM IDB leads to an increase of the resting heart rate of *ste* ^−/−^ mutants from 81 ± 1.8 to 117 ± 2.1 bpm (*n* = 10, *p* < 0.0001), indicating partial rescue of bradycardia in *ste* ^−/−^ mutants via enhanced mitochondrial function. In summary, these findings implicate an essential role of DLST for zebrafish heart rate control via ATP production.

Interestingly, as shown above, reduced ATP levels mediated by loss of DLST function, seem not to directly impact on ventricular cardiomyocyte contractile function, since fractional shortening of *ste* ^−/−^ mutants is not reduced under unstressed conditions, in comparison to the wild-type siblings. Hence, to evaluate if enhancement of ATP consumption might unmask the expected effect of ATP reduction on cardiomyocyte contractile function, we next raised the offspring of *ste* heterozygous embryos at an increased temperature of 32 °C [29, 32]. As shown in Fig. 5c, under normal rearing conditions of 29 °C, *ste* ^−/−^ mutant embryos exhibit preserved cardiac systolic function throughout the first 5 days of embryonic development, whereas raising the offspring at 32 °C unmasks impaired systolic ventricular function in *ste* ^−/−^ mutants. After 72 hpf ventricular fractional shortening is reduced to 48 ± 2.1 % in offspring raised at 32 °C compared to 54 ± 1.7 % in *ste* ^−/−^ mutants raised at 29 °C (*n* = 10, *p* = 0.0358), at 96 hpf down to 18 ± 4.0 % compared to 58 ± 1.8 % (*n* = 10, *p* < 0.0001) and at 120 hpf down to 9.9 ± 3.5 % compared to 51 ± 2.8 % (*n* = 10, *p* < 0.0001) in embryos raised at 29 °C.

### Loss of DLST leads to pacemaker cell dysfunction in *ste*^−/−^ mutant zebrafish

**Fig. 6:**
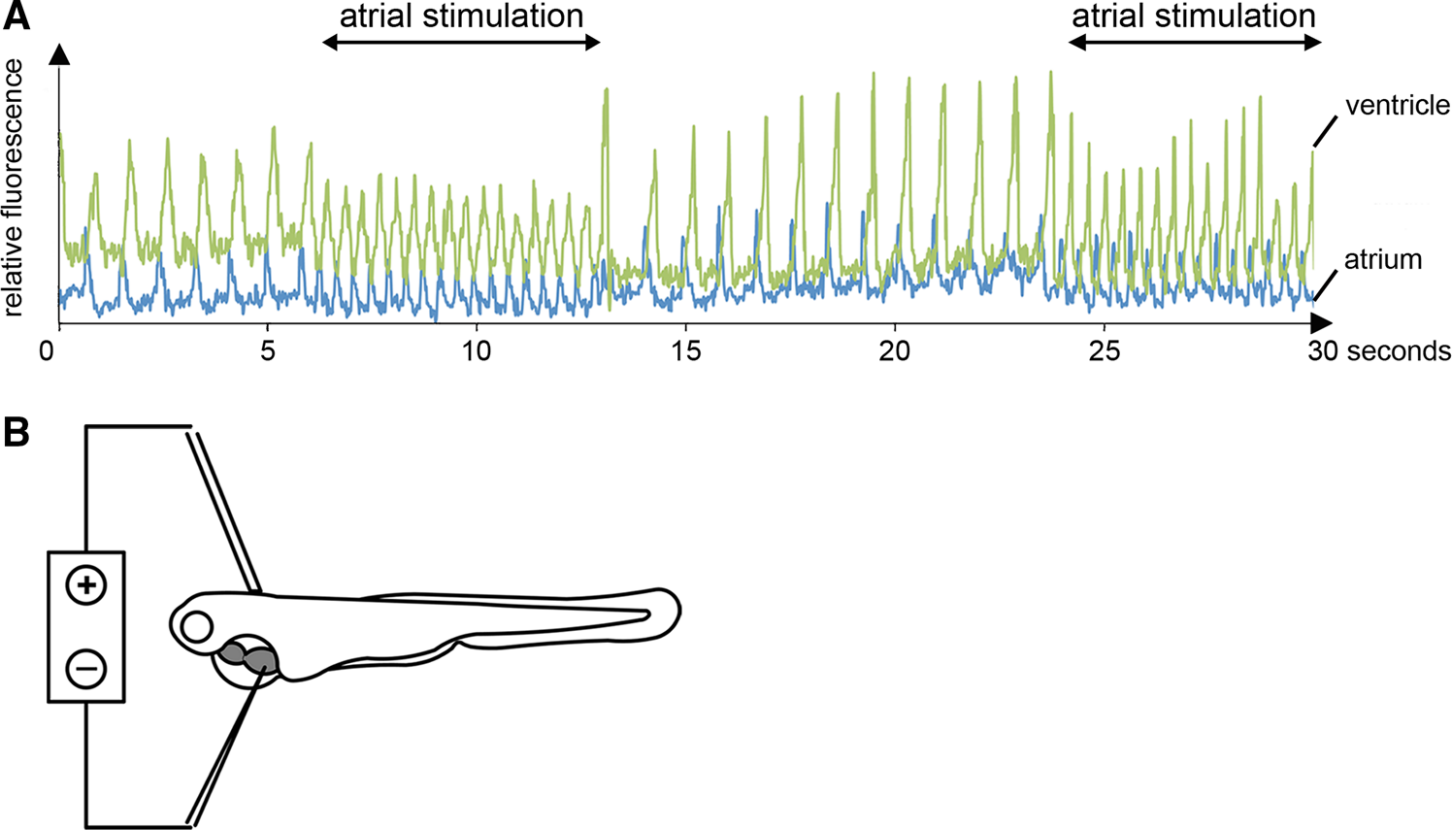
**a** Selective atrial stimulation of *ste* ^−/−^ mutants at 72 hpf reconstitutes heart rate to normofrequency as depicted by simultaneous measurements of atrial (*blue*) and ventricular (*green*) cytoplasmic calcium transients. **b** Schematic description of the experimental setup for selective atrial stimulation using an electric stimulator

The observed reduced resting heart rate in *ste* ^−/−^ mutants might be either caused by defective excitation generation in the pacemaker site of the heart, the so-called sinus node, by slowed-down myocardial excitation propagation in the atrium and the ventricle, or by uncoupling of excitation and contraction. To decipher the underlying pathophysiology of *ste* ^−/−^ bradycardia, we next assayed cardiac calcium transients [23] and performed external electrical pacing experiments. First, to evaluate if abnormal cardiac excitation propagation or excitation–contraction coupling account for the *ste* phenotype, we monitored free cytosolic myocardial calcium and cardiac contraction simultaneously in *ste* ^−/−^ mutant hearts at 72 hpf. As in wild-types, cardiac excitation in *ste* ^−/−^ mutant embryos starts in the atrium, and then moves from the atrium towards the ventricle. Each atrial calcium wave is followed by a calcium wave through the ventricle and each calcium wave is accompanied by cardiac chamber contraction (*n* = 15/15, exemplary Supplementary Movie), indicating that cardiac excitation in *ste* ^−/−^ mutants is correctly initiated in the atrium, but with a much lower frequency compared to wild-types (Fig. 6a).

Next, to evaluate if either slowed excitation initiation in the cardiac pacemaker cells, or a prolonged electrical refractory period of cardiomyocytes underlies the *ste* bradycardic phenotype, we performed selective cardiac stimulation in *ste* ^−/−^ mutant embryos (Fig. 6b). As shown in Fig. 6a, similar to the situation in wild-type hearts, selective electrical atrial stimulation above the endogenous increases the heart rate in *ste* ^−/−^ mutants from around 80 up to 150 bpm (*n* = 15, exemplary Supplementary Movie), whereby each atrial stimulation is followed by coordinated sequential atrial and ventricular contractions. In summary, these findings indicate that bradycardia in *ste* ^−/−^ mutants is caused by defective excitation initiation in atrial pacemaker cells, so-called sinus bradycardia, and not by defective excitation propagation or excitation–contraction uncoupling.

## Discussion

In search for genetic regulators of the vertebrate heart rate, we isolated in a large-scale mutagenesis screen the bradycardic zebrafish mutant *schneckentempo* (*ste*) and find here by positional cloning that loss of *dihydrolipoyl succinyltransferase* (DLST) function leads to impaired cardiac pacemaker function in *ste* ^−/−^ mutant embryos. DLST is an essential component of the α-ketoglutarate dehydrogenase complex of the citric acid cycle localized in mitochondria, and hence involved in ATP production. Accordingly, we find severely altered mitochondrial morphology and reduced ATP levels in *ste* ^−/−^ mutant zebrafish embryos, suggesting that limited energy supply is the molecular cause for the observed bradycardia in *ste* ^−/−^ mutants.

The essential role of regular ATP production and normal ATP levels for heart rate regulation of the embryonic zebrafish heart is further confirmed by the pharmacological inhibition of ATP production with the citric acid cycle blocker sodiumfluoroacetate SFA and the respiratory chain reaction uncoupler dinitrophenol DNP. Incubation of wild-type zebrafish embryos with either SFA or DNP significantly reduces global ATP levels in the zebrafish embryos as observed in the *ste* ^−/−^ zebrafish mutants and causes reduced resting heart rates.

In contrast to this, resting heart rate in *ste* ^−/−^ mutants can partially be rescued by incubation with the coenzyme Q10 analogue Idebenone, which is known to promote ATP production and improve mitochondrial function [6, 19], thereby further confirming that the effect of loss of DLST on resting heart rate is mainly mediated via ATP.

Cardiac calcium transient measurements and external electrical pacing experiments reveal that under normal conditions loss of DLST rather selectively impacts on cardiac pacemaker cell function, whereas contractile force is not significantly altered in *ste* ^−/−^ mutants, although ATP levels are globally reduced. However, enhancement of ATP consumption by rearing *ste* ^−/−^ mutants at higher temperatures unmasks an impact of DLST also on ventricular contractile function. DLST is known to catalyze energy generation in the citric acid cycle in the mitochondrial matrix, where GTP, NADH and FADH_2_ are produced. The energy equivalents NADH and FADH_2_ are then converted to ATP in the respiratory chain complex in the mitochondrial intermembrane space. Normally, this oxidative phosphorylation accounts for more than 90 % of the energy production of cells under normoxic conditions [14, 17, 35, 43]. Patients suffering from reduced activity of DLST develop lactate acidosis and usually die early during childhood. Similar to *ste* ^−/−^ mutants, cardiac morphology appears to be unaltered in DLST-deficient patients [5, 18, 26]. However, all reported cases in humans feature a residual DLST activity of 5–25 % [5, 18, 26]. By contrast, we expect no residual activity of DLST in *ste* ^−/−^ mutant zebrafish since translation of DLST is predicted to be prematurely terminated and significant nonsense mediated RNA decay of the mutated *dlst* transcript is observed in *ste* ^−/−^ mutants.

Disorganization of the mitochondrial inner membrane and deranged architecture of cristae are common in human disorders featuring mitochondrial dysfunction [51]. Deficiency of the citric acid cycle is known to increase production of free radicals thereby inducing mitochondrial damage [35]. Similar to the situation in humans [25], we find severe alterations in mitochondrial morphology in the hearts and skeletal muscle of *ste* ^−/−^ mutants with disorganization and increased mitochondrial density as well as vacuoles within mitochondria and in their vicinity. The *ste* ^−/−^ mitochondria exhibit an irregularly shape with reduced cristae.

Interestingly, in a retrospective analysis of 90 patients with inherited mitochondriopathies, 22.2 % exhibited cardiac arrhythmias including sinus bradycardia [49]. ATP levels are significantly reduced in *ste* ^−/−^ mutants to one-third of the ATP content of wild-type zebrafish embryos. In the DLST-deficient *ste* ^−/−^ mutant embryos ATP production via oxidative phosphorylation is supposedly disrupted. However, ATP can usually also be produced by alternative pathways such as anaerobic glycolysis [14]. Furthermore, ATP can be provided by maternally inherited mitochondria. Similar to the situation in humans, maternal inheritance of the mitochondrial DNA and passing on of thousands of functionally active maternal mitochondria to the embryo warrants oxidative phosphorylation during zebrafish embryogenesis [3, 16, 41, 50]. Interestingly, bradycardia in the *ste* ^−/−^ mutant zebrafish is first present at 72 hpf. At earlier developmental stages, we find no significantly reduced heart rates in *ste* ^−/−^ mutant embryos, suggesting that at these stages the residual ATP production supposedly by maternal mitochondria is still sufficient to maintain a normal heart rate.

There are three different possibilities how these reduced ATP levels in *ste* ^−/−^ mutants might lead to bradycardia. Reduced ATP levels could either impact on: (1) myofilament activation, (2) excitability of the working myocardium or (3) the depolarization of cardiac pacemaker cells. However, we find here by external electrical stimulation experiments and calcium transient analyses that bradycardia in *ste* ^−/−^ mutants is not caused by prolonged refractoriness of cardiomyocytes to excitation, or by uncoupling of cardiomyocyte excitation–contraction, but rather by reduced firing rates of the cardiac pacemaker cells.

The pacemaker site of the zebrafish heart is localized in the right dorsal quadrant of the atrium, near the inflow tract of the atrium and the sinus venosus [2]. Tessadori et al. [44] demonstrated that the pacemaker cells in zebrafish resemble those in mammals in their gene expression pattern and electrochemical properties, in particular their ability to spontaneously depolarize. In mammals, a variety of regulatory mechanisms exist to influence the action potential frequency in sinus node cells [40, 48]. Pacemaker cells in mammals depend strongly upon ATP to function and to regulate their activity [48]. Yaniv et al. [48] recently demonstrated that in pacemaker cells the ATP supply is closely matched to the demand and is rapidly adjusted in phases of high ATP demand. ATP is necessary for the regulation of the action potential frequency in these cells via the Ca^2+^–calmodulin activated adenylyl cyclase/proteinkinase A, Ca^2+^–calmodulinkinase II, cAMP and Ca^2+^ pathways. The reduced ATP levels in *ste* ^−/−^ mutants are expected to impact on cAMP levels, since ATP is necessary to activate adenylyl cyclase to produce cAMP [48]. Interestingly, absence of cAMP in cardiac pacemaker cells is known to cause sinus bradycardia in mammals [40]. However, regular mitochondrial function not only warrants ATP production and enables consecutive pathways such as the cAMP/Ca^2+^ pathway, but is also crucial for various other essential mechanisms and metabolic pathways in mammals [1, 13]. Therefore, other ATP-independent mechanisms apart from reduced ATP levels might partially contribute to cardiac dysfunction in *ste* ^−/−^ mutant zebrafish embryos, since the *ste* ^−/−^ mutants’ mitochondrial function and structure is severely disrupted. Mitochondria are both an essential source of ROS as well as an important target of injury by ROS [25, 39]. Therefore, mitochondriopathies are often accompanied by enhanced ROS production causing further mitochondrial damage and leading to cell damage and inducing apoptosis [25, 39]. Similarly, increased ROS production and its consecutive injury to cells due to loss of mitochondrial DLST function possibly contribute to cardiac dysfunction in *ste* ^−/−^ mutants and thereby impact on heart rate regulation.

In search for novel genetic heart rate regulators den Hoed and co-workers recently conducted a GWAS meta-analysis and linked 14 chromosomal loci to heart rate regulation [12]. Interestingly, one of the associated loci contains DLST. Hence, thorough genetic analysis might reveal a modifying or even causal role of DLST gene variants in the pathogenesis of cardiac pacemaker diseases, such as the sick sinus syndrome.

## Electronic supplementary material

Below is the link to the electronic supplementary material.

**Supplementary Fig.** **1** Human and zebrafish DLST are highly homologous. Amino acid sequence alignment of human (hdlst), zebrafish (zdlst) and *ste* ^−/−^ mutant DLST. Human and zebrafish DLST share 74 % amino acid identity. The highly conserved lipoyl and catalytic domains are indicated with red lines above the alignment (TIFF 485 kb)

**Supplementary Movie** Electrical stimulation of a *ste* ^−/−^ mutant heart at 72 hpf. *ste* ^−/−^ mutants were injected with fluorescent dye to monitor free cytosolic myocardial calcium and cardiac contraction simultaneously. Electrical stimulation drives up basal heart rate of *ste* ^−/−^ mutants to normofrequency and leads to sequential and coordinated contractions of atrium and ventricle (MPG 7736 kb)
