# Supplementary figures and images for: Loss of dihydrolipoyl succinyltransferase (DLST) leads to reduced resting heart rate in the zebrafish
Source: Basic Res Cardiol. 2015 Feb 20;110(2):14. doi: 10.1007/s00395-015-0468-7 (PMC4335124; doi:10.1007/s00395-015-0468-7)

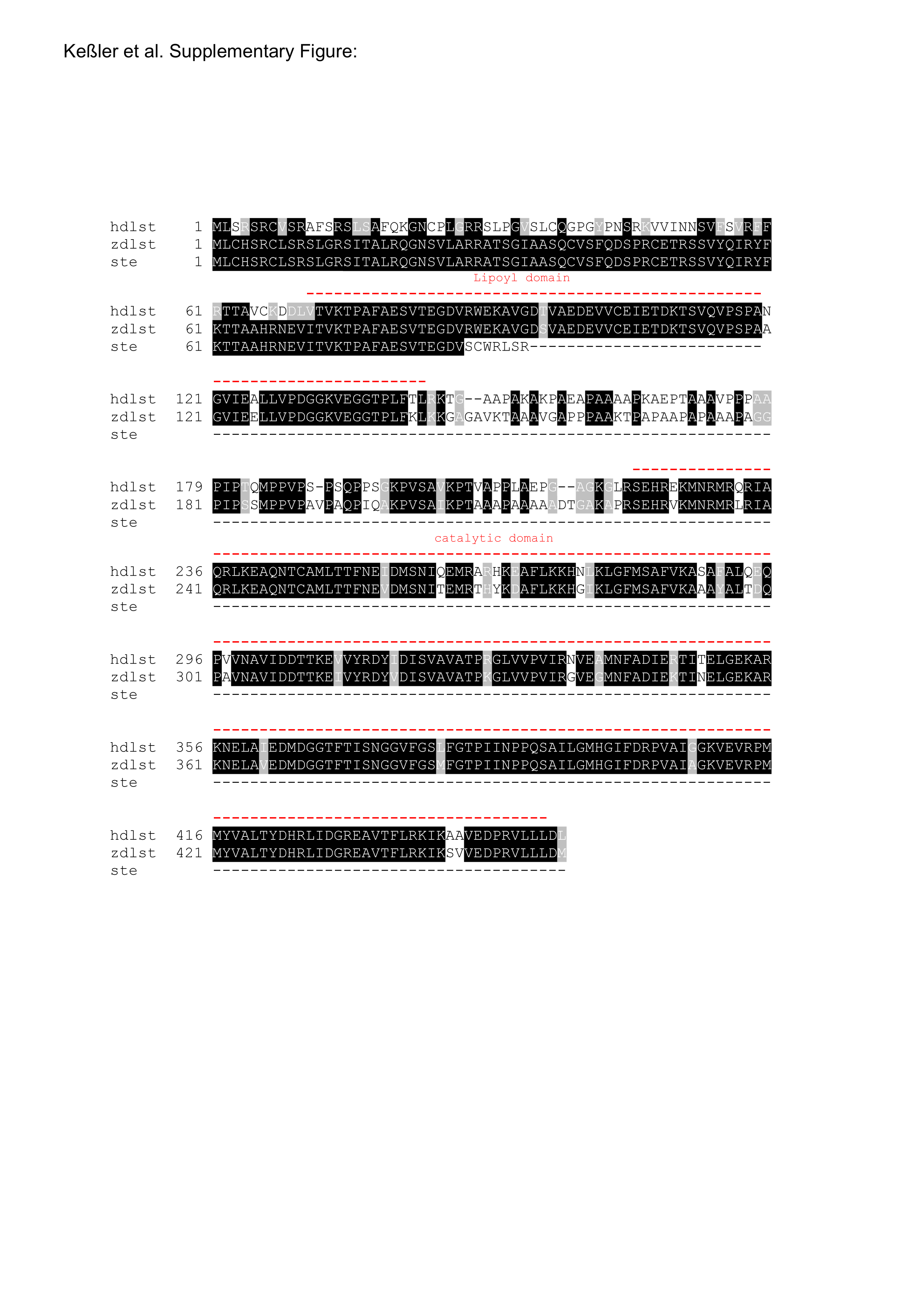

Supplement: Supplementary file 1 — Supplementary Fig. 1 Human and zebrafish DLST are highly homologous. Amino acid sequence alignment of human (hdlst), zebrafish (zdlst) and ste −/− mutant DLST. Human and zebrafish DLST share 74 % amino acid identity. The highly conserved lipoyl and catalytic domains are indicated with red lines above the alignment (TIFF 485 kb) [file 395_2015_468_MOESM1_ESM.tiff]
